# Supplementary material for: VOx Phase Mixture of Reduced Single Crystalline V2O5: VO2 Resistive Switching
Source: Materials (Basel). 2022 Oct 31;15(21):7652. doi: 10.3390/ma15217652 (PMC9653758; doi:10.3390/ma15217652)
Supplement: Supplementary file 1 [file materials-15-07652-s001.zip › SI.pdf]

VO_x phase mixture of reduced single crystalline V_2O_5: VO_2 resistive switching

B. Walls, O. Murtagh, S. I. Bozhko, A. Ionov, A. A. Mazilkin, D. Mullarkey, A. Zhussupbekova, D. Shulyatev, K. Zhussupbekov, N. Andreev, N. Tabachkova, I. V. Shvets

Supplemental Information

1 Reduction of $V_2O_5(010)$

Figure S1 depicts the full range θ-2θ scan of the annealed crystal discussed in length in the manuscript.

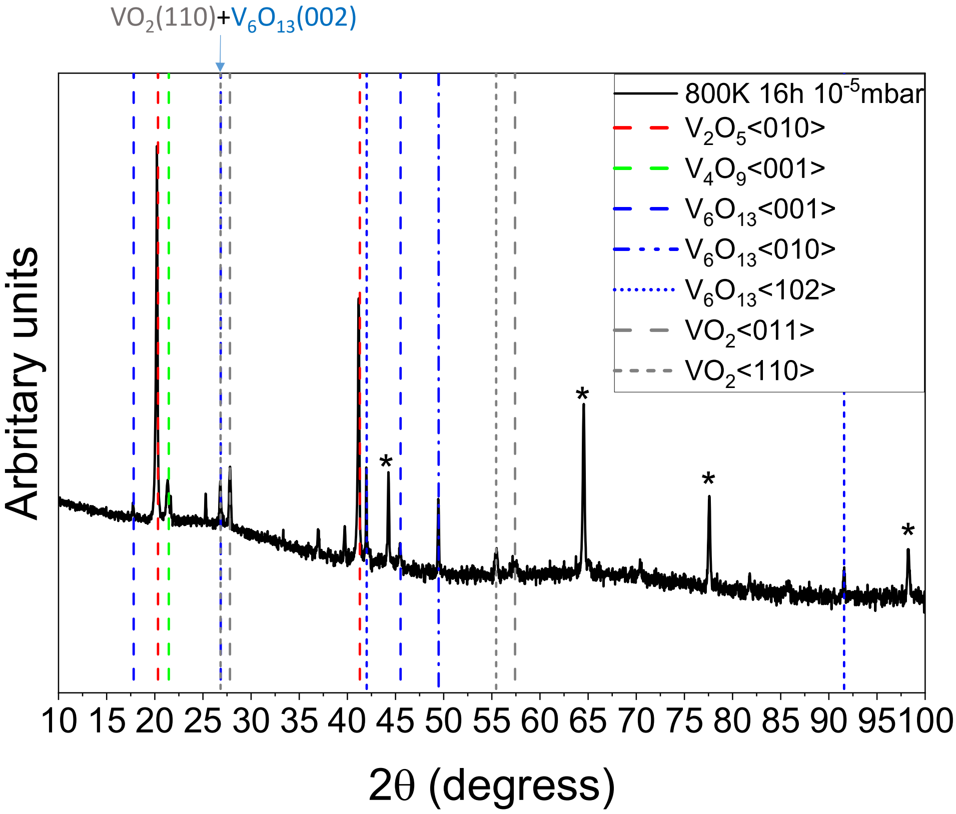

Figure S1: Full XRD scan of the annealed crystal discussed in the main text. The crystal has been annealed at 800 K for 16 h in a pressure of 10^{-5} mbar. Asterisks represent peaks corresponding to the sample stage. Note that at 27° a $V_6O_{13}(001)$ and $VO_2(110)$ peak are overlapping. V_2O_5 [24], V_4O_9 [25], V_6O_{13} [35] and VO_2 [34]

The reduction process has been examined as a function of vacuum anneal temperature. This informed the annealing conditions of the crystal examined in detail in the main text. Resistance measurements of crystals vacuum annealed at 600, 700, 800 and 900 K are depicted in Figure S2. In Figure S3 the XRD θ-2θ scans of the crystals are depicted.

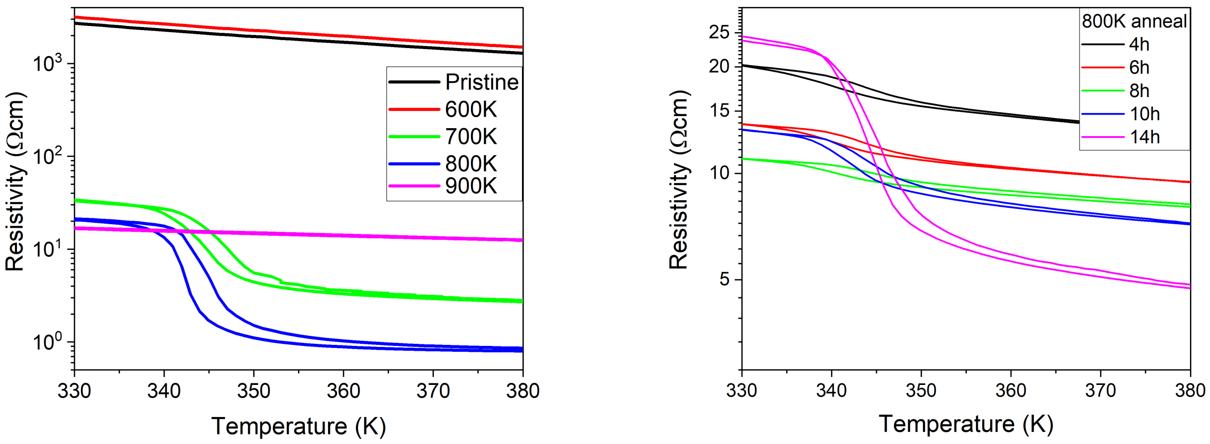

Figure S2: Left: resistance measurements of the crystals annealed at different temperatures. In each case further annealing only induces minor changes in the resistance profile. Right: resistance measurements of a crystal annealed at 800K for different durations.

The large resistance of 600 K annealed crystal suggests V_4O_9 - the only additional phase introduced by the anneal - is of comparable resistance to V_2O_5. The lack of a VO_2 resistance change in the 900K annealed crystal is suggested to be due to V_6O_{13} dominating the crystal, as evidenced by the XRD.

The evolution of the resistance of a crystal annealed at 800 K is depicted in Figure S2 right panel. After several hours of annealing a broad and small VO_2 transition is apparent (—). With further annealing the resistance decreases over the entire temperature range, while the VO_2 transition characteristics are largely unchanged (— and —). This is consistent with an increased content of metallic V_6O_{13}, which is the only metallic phase identified. Further annealing increases the resistance at low temperatures and decreases the resistance at higher temperature, and hence increases the magnitude of the VO_2 resistive switch at around 345 K (— and —). This is consistent with an increased VO_2 weight percent, which is higher (lower) resistance compared to V_6O_{13} at low (high) temperatures.

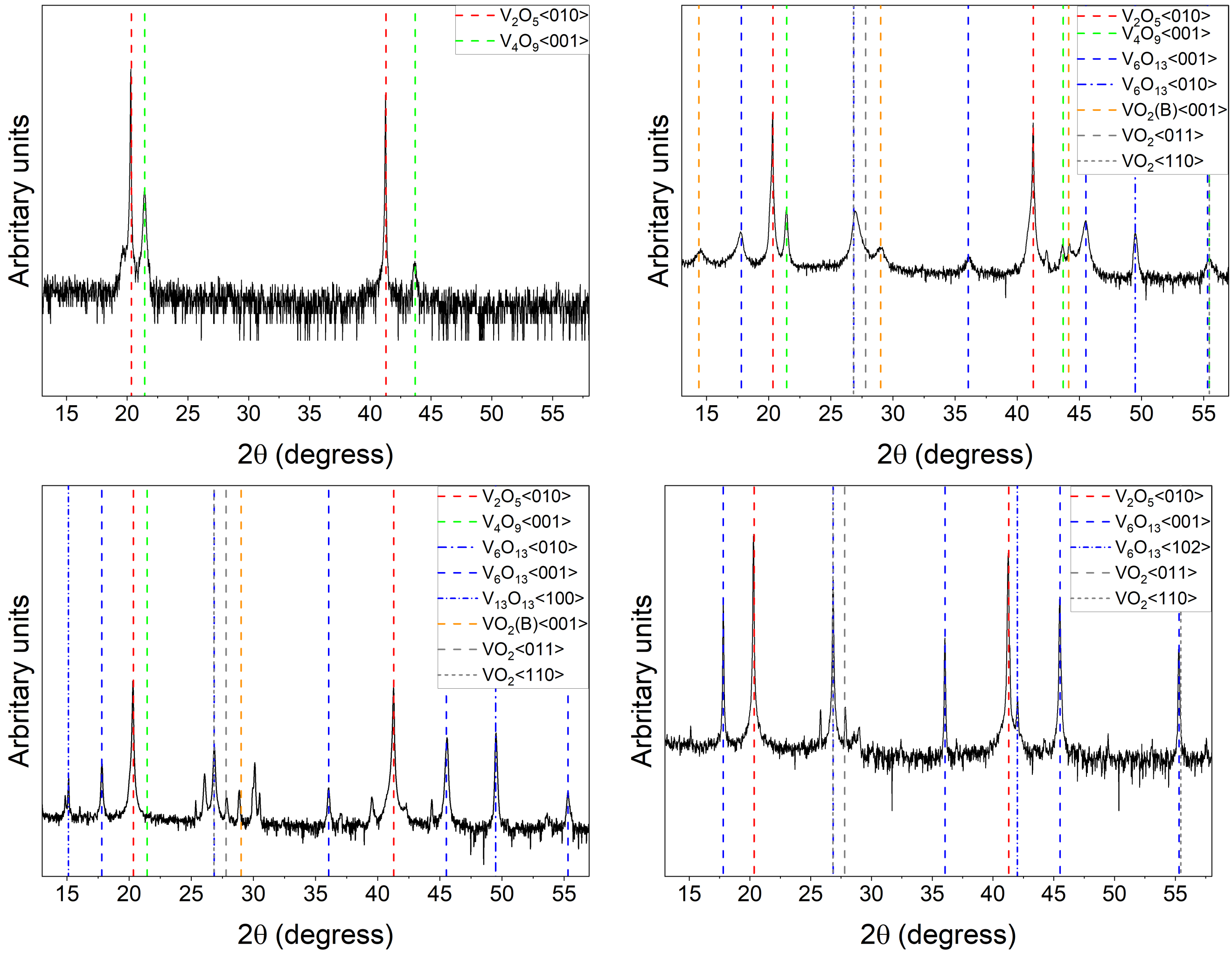

Figure S3: V_2O_5 crystals have been annealed at different temperatures; 600 (top left), 700 (top right), 800 (bottom left) and 900 K (bottom right). The pressure was 10^{-5} mbar in each case. Note that at 27° a V_6O_{13}(001) and VO_2(110) peak are overlapping. V_2O_5 [24], V_4O_9 [25], V_6O_{13} [35], VO_2(B) [28] and VO_2 [34]

2 Reproducibility of electrical measurements

For the voltage-current (VI) measurements discussed the manuscript the VI was measured 6 times at each set temperature. The left top panel of Figure S4 depicts the 30 VI measurements, 6 at each set temperature. No appreciable degradation of the VI characteristics are observed. Inset depicts 20 consecutive VI measurements at 341 K. Again, no appreciable degradation of the VI characteristics are observed. Several weeks after these measurements the same crystal was remeasured; the IV characteristics at 340 K are shown in the top right panel of Figure S4. The low voltage resistive switching is observed. The VI was measured 6 times.

The bottom left panel depict three different crystals annealed under the same conditions (800 K in a pressure of 10^{-5} mbar) with the exception of duration. Crystal 1 (black) corresponds to a crystal annealed for 8 h (---) and

subsequently for another 8 h (—, 16 h total). Crystal 2 (red) corresponds to a crystal annealed for 26 h in 2 hour steps. Crystal 3 (green) corresponds to a crystal annealed for 16 h continuously (crystal examined in the paper). We consistently observe the VO_2 transition and a low resistance at temperatures below the VO_2 transition temperature. The differences are certainly related to the duration of the anneal and the correlated progression of the reduction. VI measurements on crystal 1 (- - -) in the bottom right panel again reveal low voltage resistive switching. The higher threshold and small resistance change - compared to the top left panel - are due to the larger resistance below the transition temperature and the small thermal resistance change, respectively.

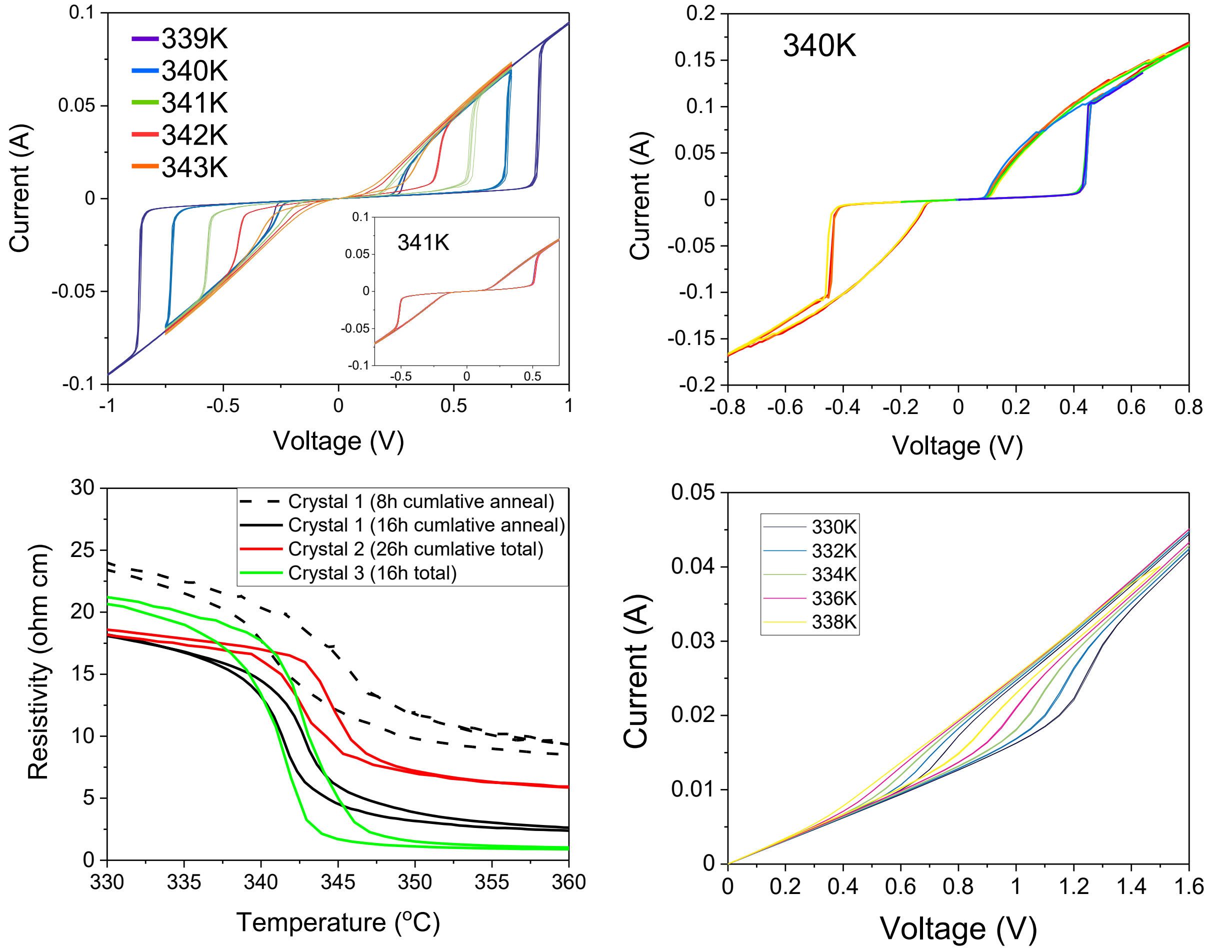

Figure S4: Cycling of the VI measurements. Left top panel; At each temperature the VI is measured 6 times. Inset are 20 measurements at 341 K. Right top panel; measurements were repeated several weeks later on a different part of the crystal. Bottom left panel; resistance of three different crystals. Right bottom panel; VI measurements of crystal 1 (- - - in bottom left panel).

3 Scanning electron microscopy

Figure S5 demonstrates the scanning electron microscopy images of the samples before focused ion beam lamella preparation for transmission electron microscopy measurements. White dashed rectangles show the orientation of the lamellae to the main sample.

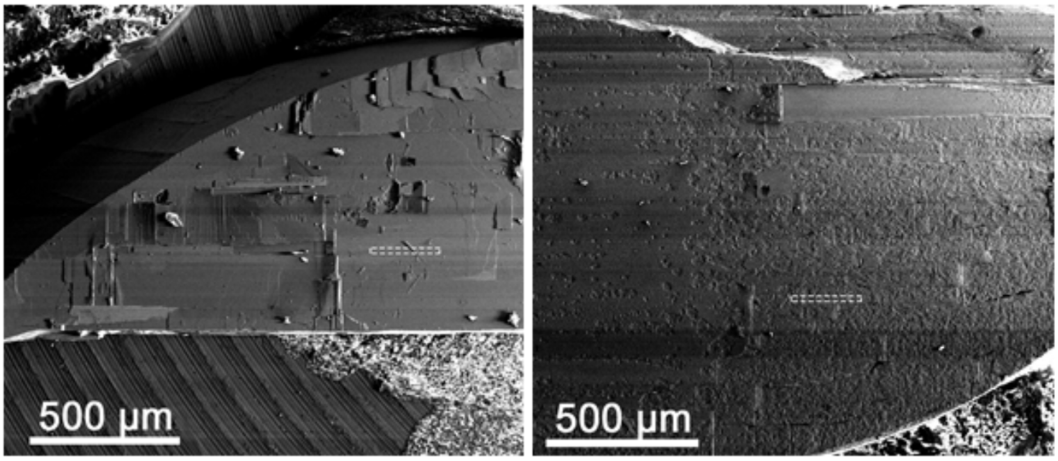

Figure S5: SEM images of the samples before TEM lamella FIB preparation. White dashed rectangles show the orientation of the lamellae to the main sample.
