# Supplementary figures and images for: VOx Phase Mixture of Reduced Single Crystalline V2O5: VO2 Resistive Switching
Source: Materials (Basel). 2022 Oct 31;15(21):7652. doi: 10.3390/ma15217652 (PMC9653758; doi:10.3390/ma15217652)

Arbitrary units

$\text{VO}_2(110) + \text{V}_6\text{O}_{13}(002)$

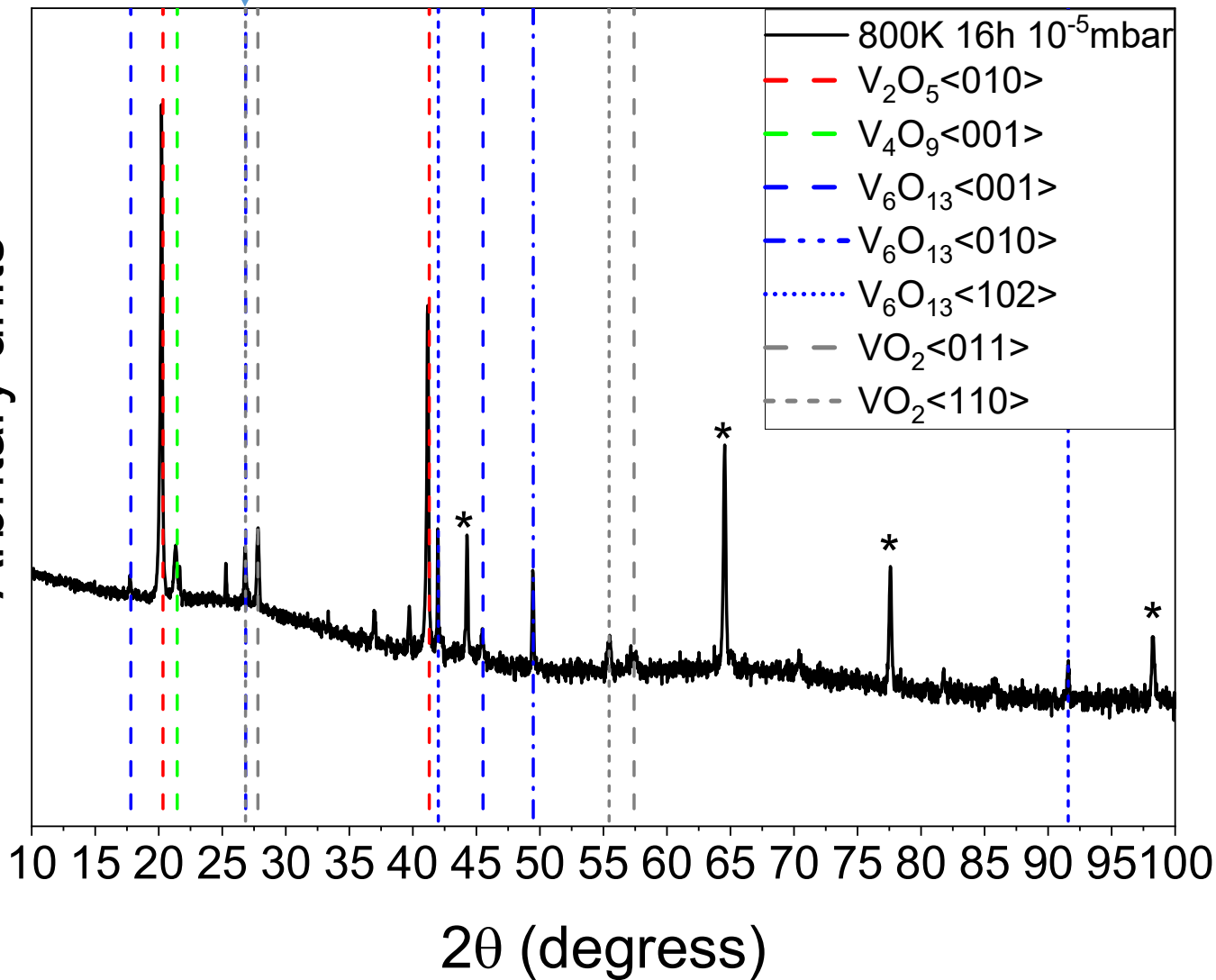

Supplement: Supplementary file 1 [file materials-15-07652-s001.zip › 1.pdf]

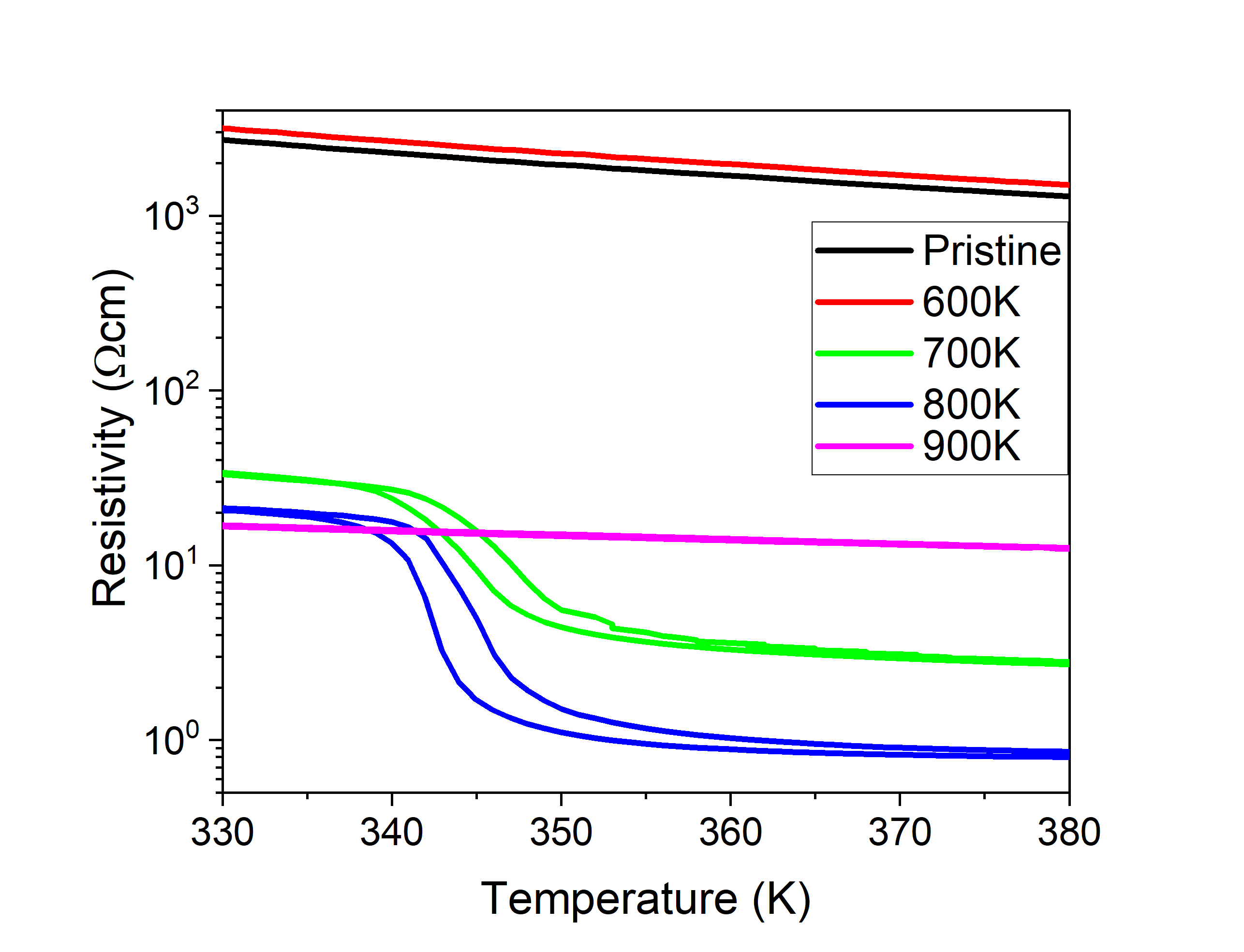

Supplement: Supplementary file 1 [file materials-15-07652-s001.zip › 2-1.png]

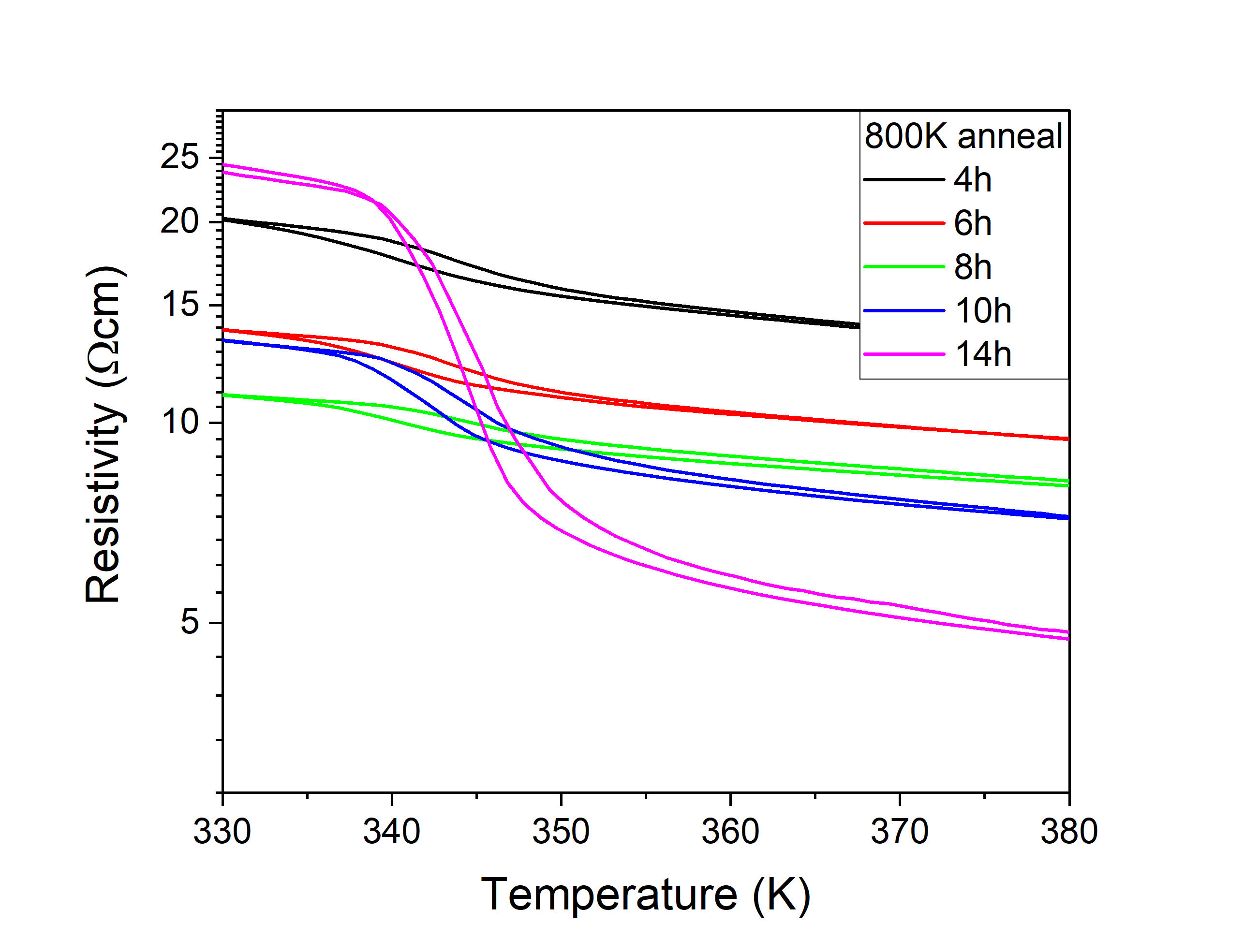

Supplement: Supplementary file 1 [file materials-15-07652-s001.zip › 2-2.png]

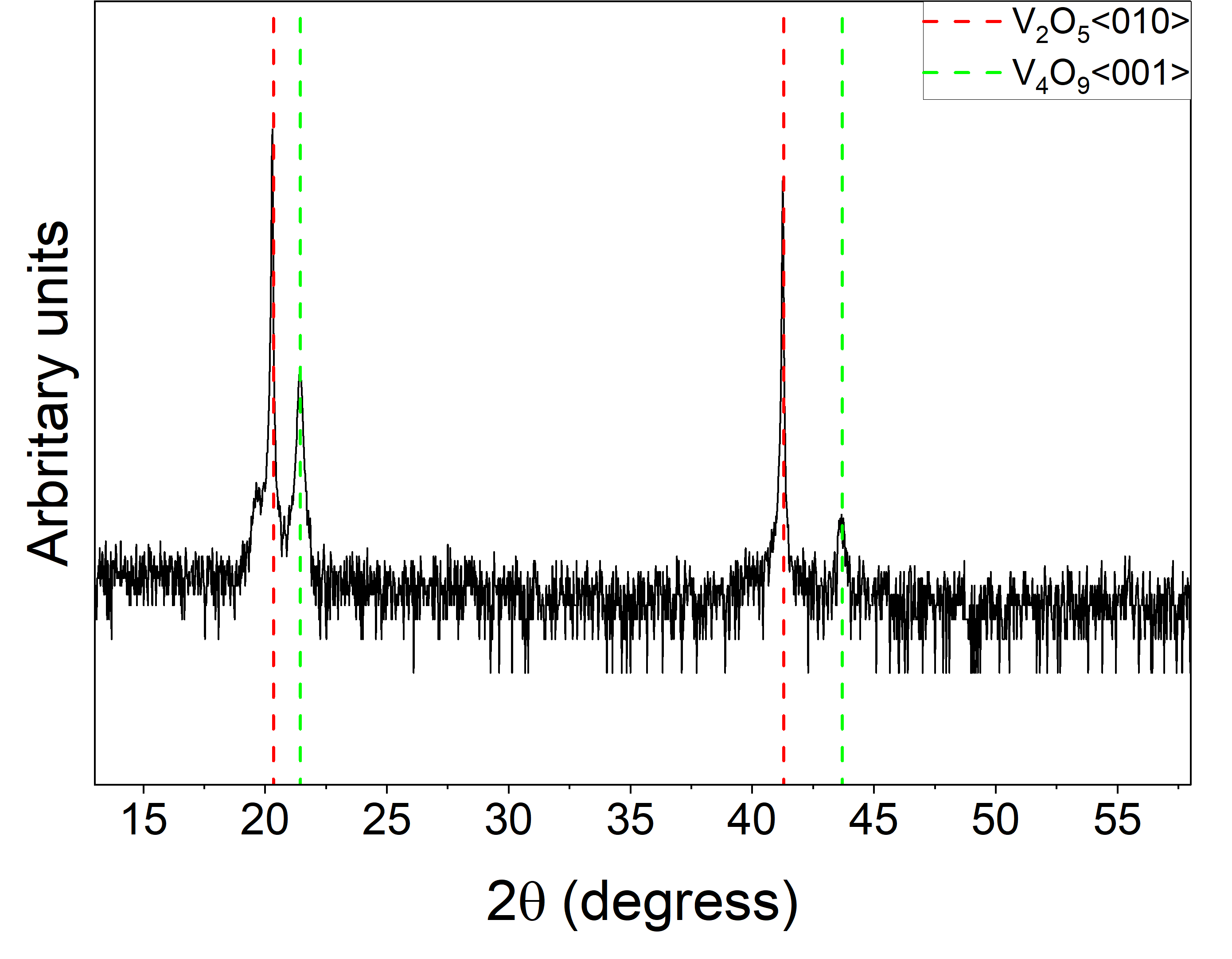

Supplement: Supplementary file 1 [file materials-15-07652-s001.zip › 3-1.png]

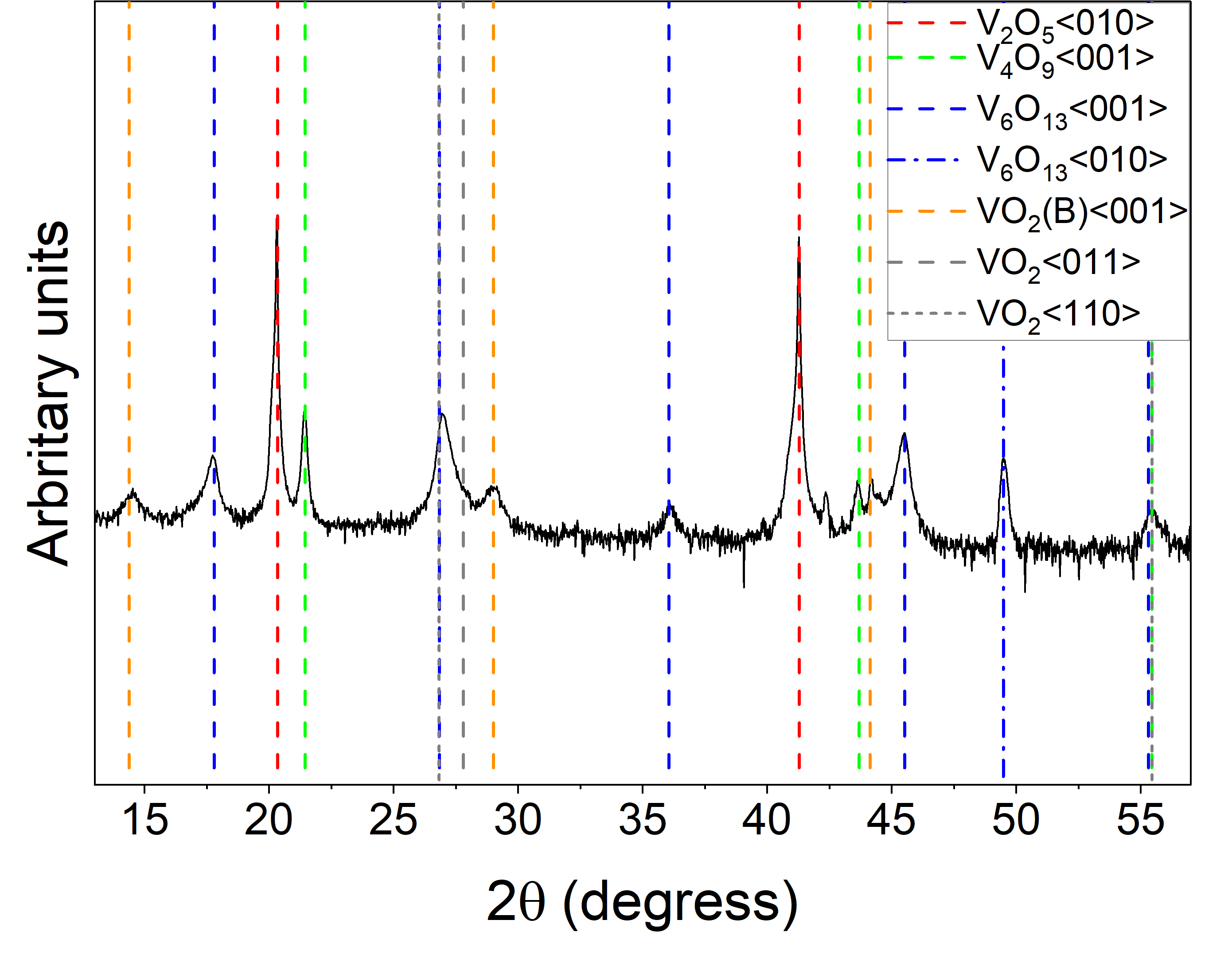

Supplement: Supplementary file 1 [file materials-15-07652-s001.zip › 3-2.png]

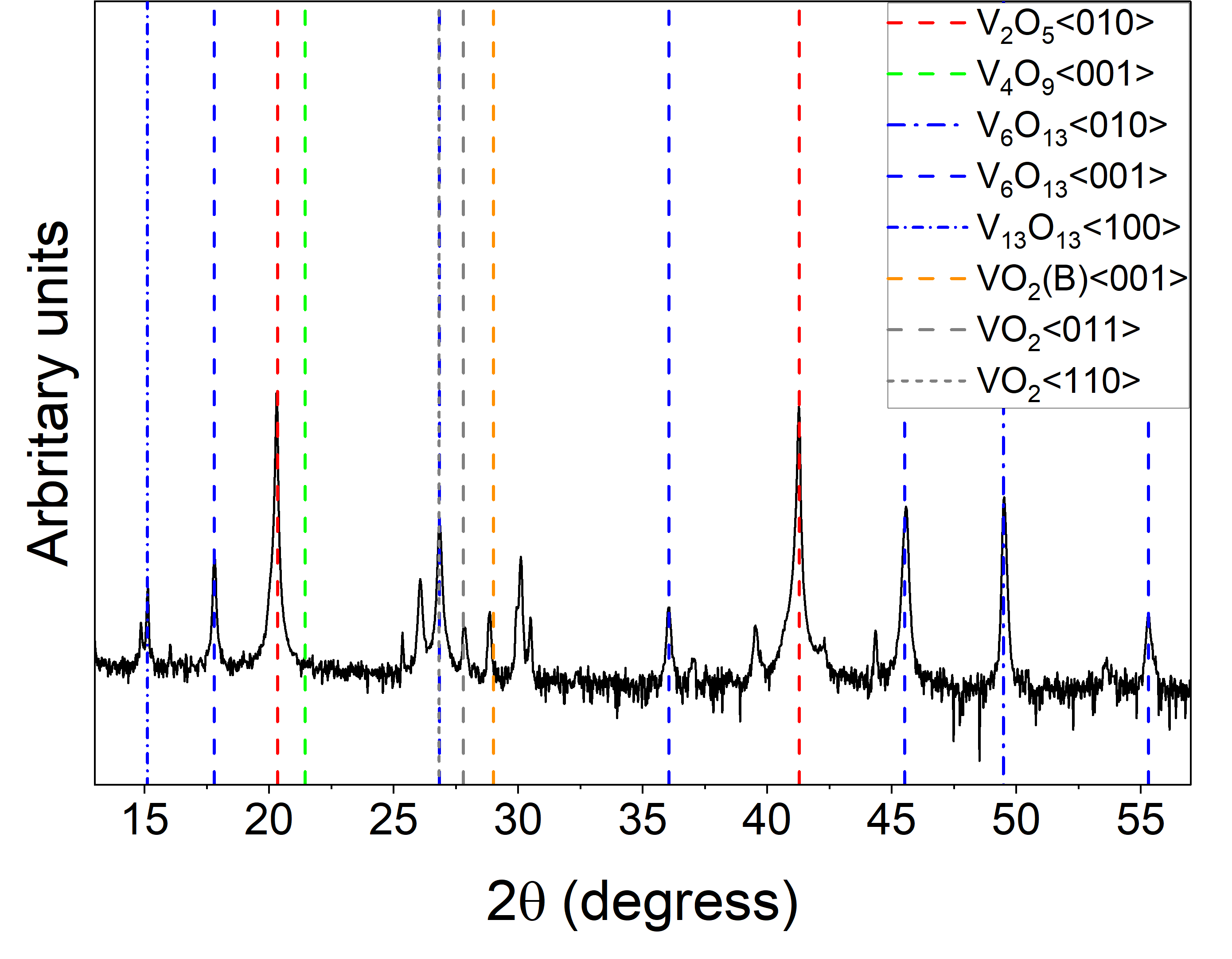

Supplement: Supplementary file 1 [file materials-15-07652-s001.zip › 3-3.png]

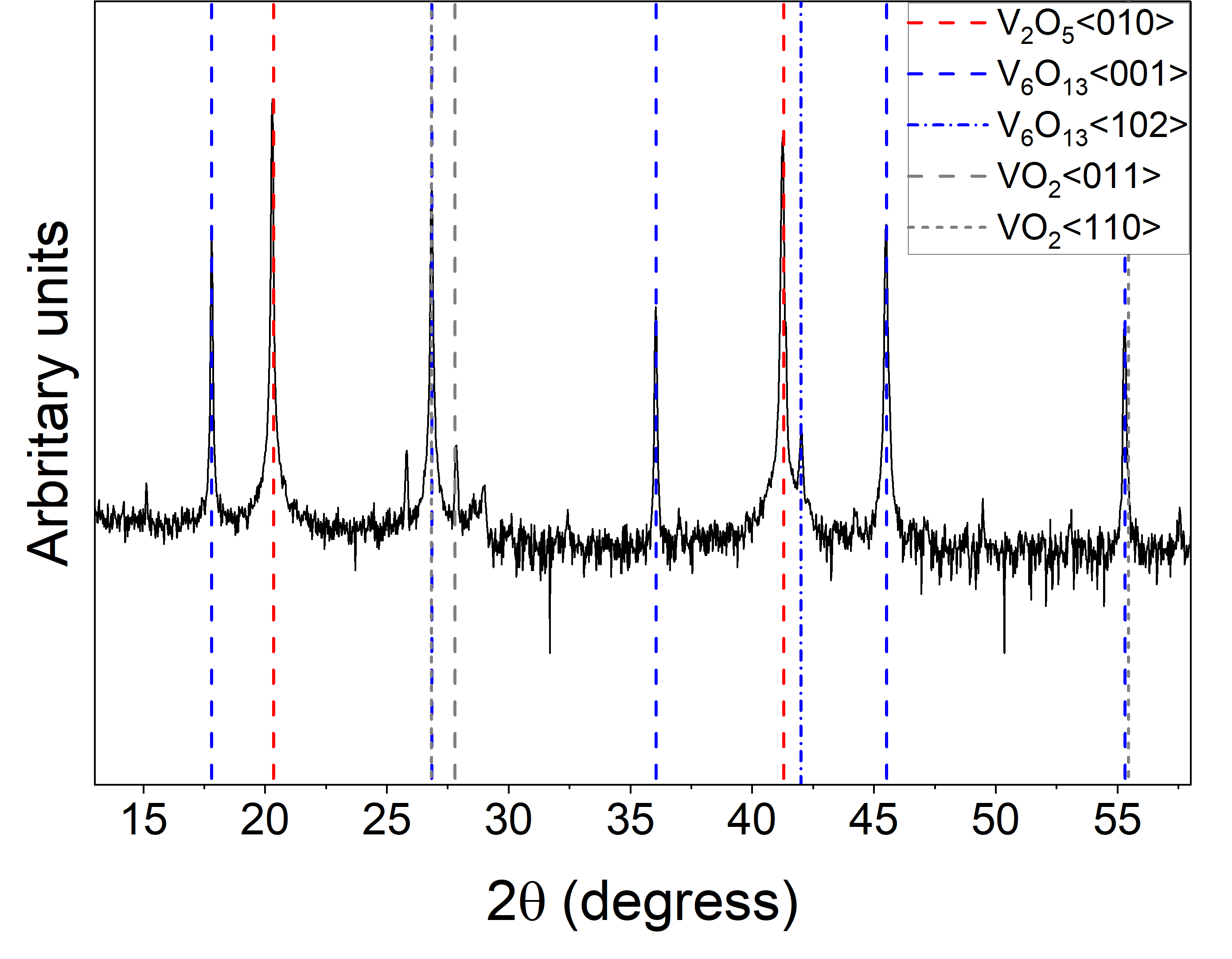

Supplement: Supplementary file 1 [file materials-15-07652-s001.zip › 3-4.png]

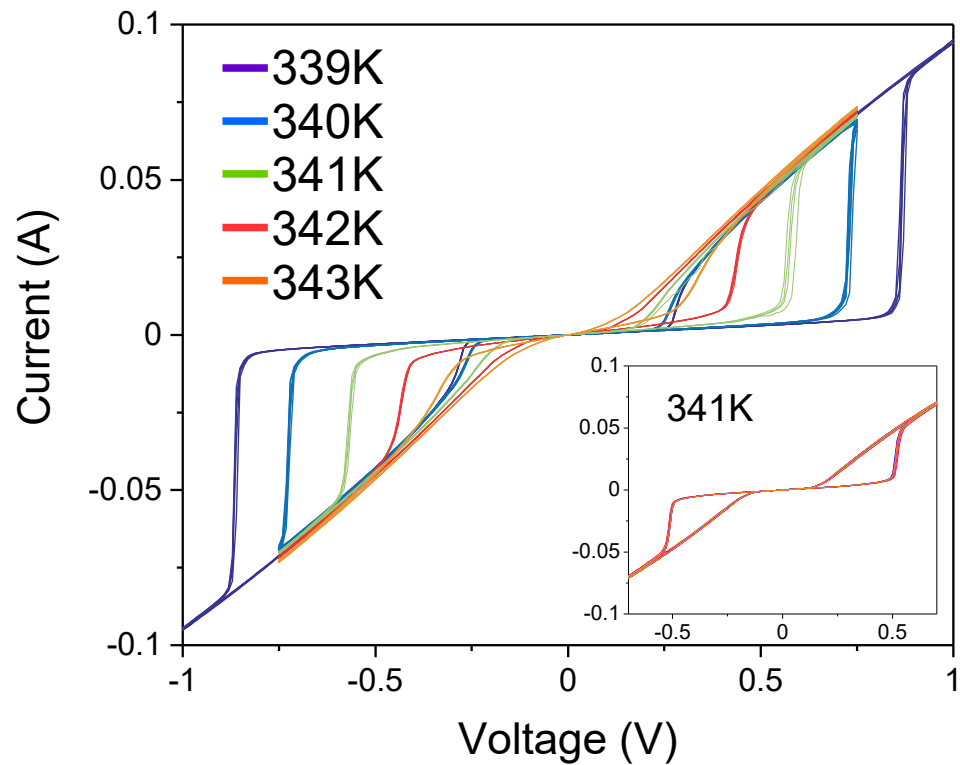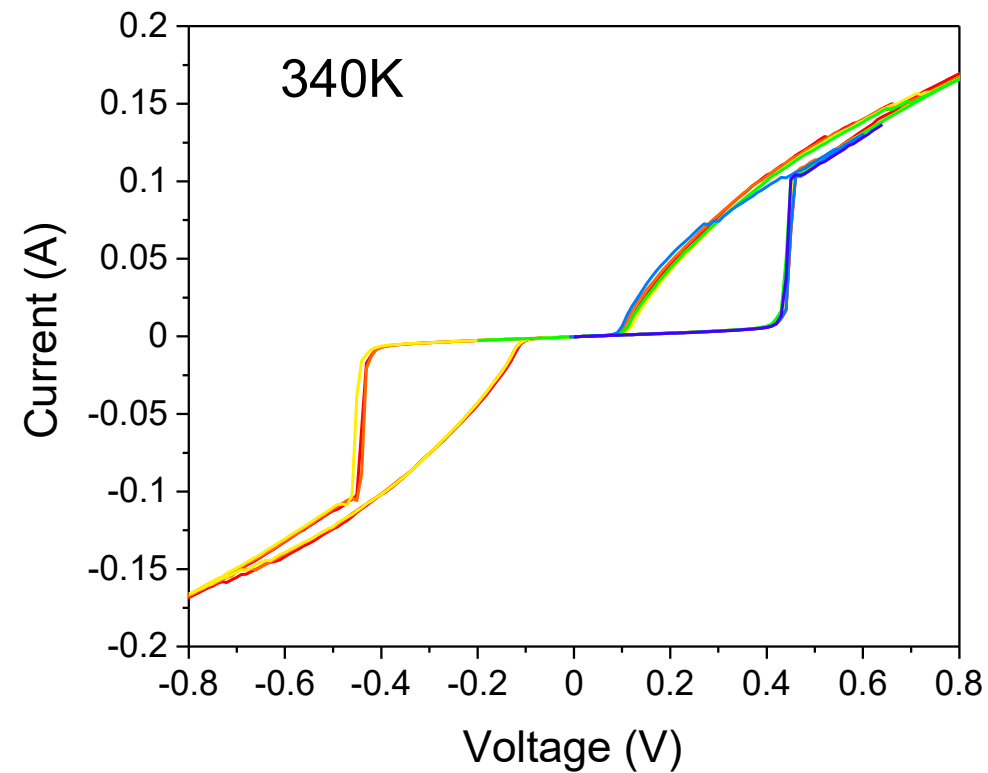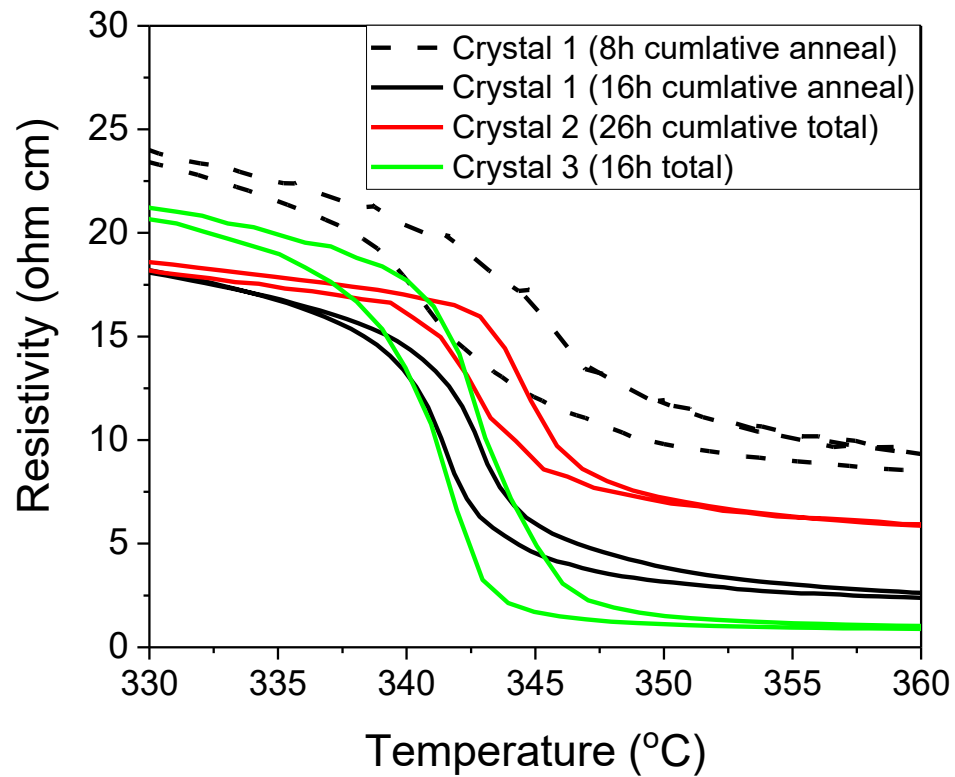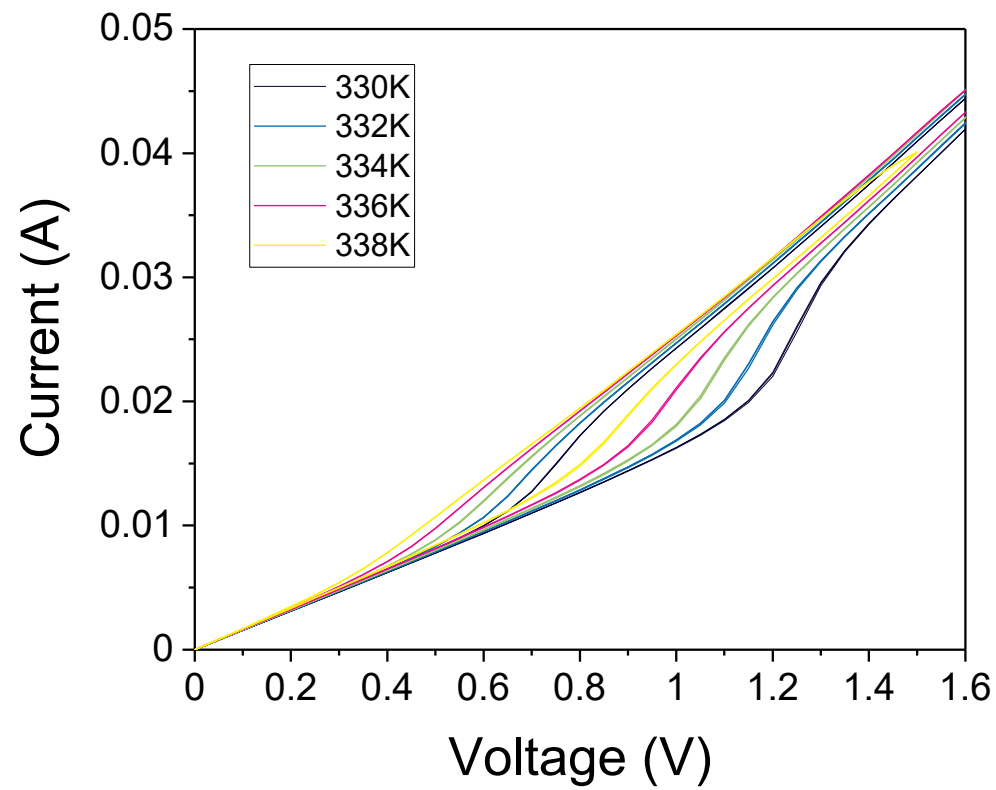

Supplement: Supplementary file 1 [file materials-15-07652-s001.zip › 4.pdf]

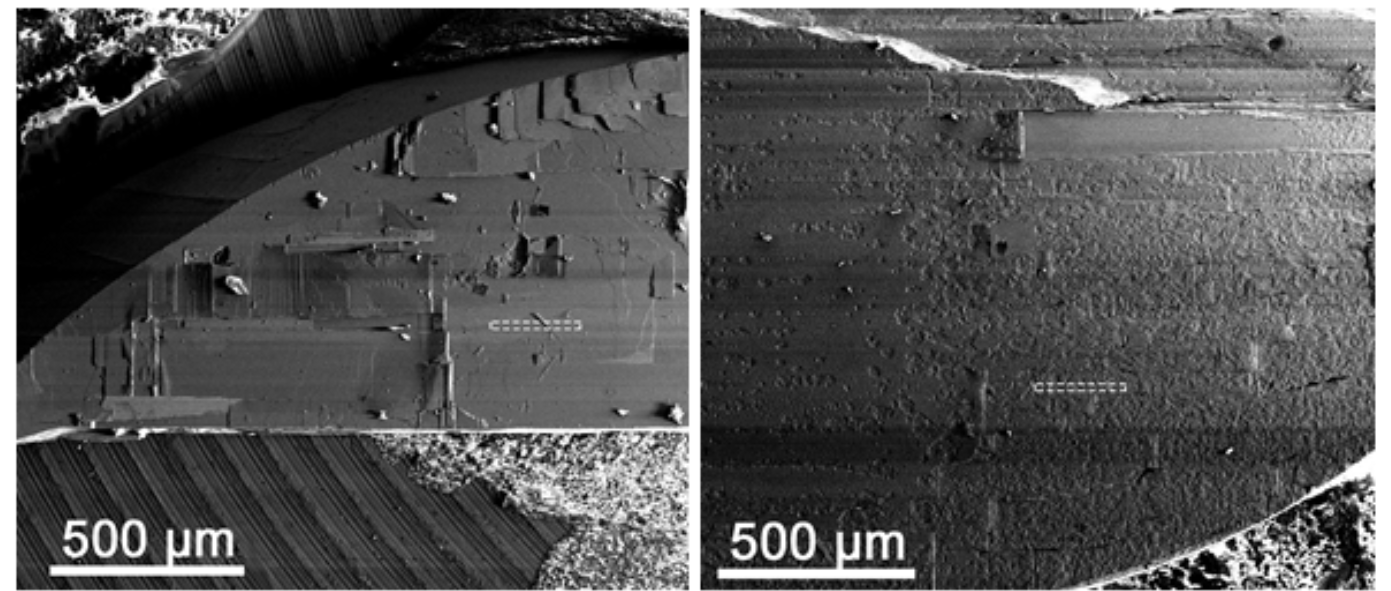

Supplement: Supplementary file 1 [file materials-15-07652-s001.zip › 5.png]
